# Evaluation of Compressive Strength and Thermal Conductivity of Sand Stabilized with Epoxy Emulsion and Polymer Solution

**DOI:** 10.3390/polym14101964

**Published:** 2022-05-11

**Authors:** Sung-Sik Park, Jun-Woo Park, Keun-Byoung Yoon, Il Seouk Park, Seung-Wook Woo, Dong-Eun Lee

**Affiliations:** 1 Department of Civil Engineering, Kyungpook National University, 80 Daehakro, Bukgu, Daegu 41566, Korea; sungpark@knu.ac.kr (S.-S.P.); geowsw@knu.ac.kr (S.-W.W.); 2 Daegu Urban Corporation, 73 Chimsanro, Bukgu, Daegu 41594, Korea; jwpark@duco.or.kr; 3 Department of Polymer Science and Engineering, Kyungpook National University, 80 Daehakro, Bukgu, Daegu 41566, Korea; kbyoon@knu.ac.kr; 4 Department of Mechanical Engineering, Kyungpook National University, 80 Daehakro, Bukgu, Daegu 41566, Korea; einstein@knu.ac.kr; 5 Department of Architecture, Civil, Environment and Energy Engineering, Kyungpook National University, 80 Daehakro, Bukgu, Daegu 41566, Korea

**Keywords:** sand stabilization, epoxy emulsion, acrylic polymer, ground-coupled heat pump, compressive strength, thermal conductivity, heat transfer fluid model

## Abstract

This paper presents findings obtained by evaluating the compressive strength, thermal conductivity, and durability of sand cylinder specimens stabilized with either epoxy emulsion (EM), acrylic polymer aqueous solution (APAS), EM-APAS mixture, or EM-APAS-lime mixture. Given the data obtained from the laboratory test, simulation analysis that uses a heat transfer fluid model of a ground-coupled heat pump (GCHP) system confirms the EM-APAS-lime binder performs best in the compressive strength and thermal conductivity; EM-APAS mixture performs best in the durability. However, the slake durability index of specimens containing EM-APAS-lime is equal to or greater than 80%. In addition, the compressive strength of sand stabilized with the EM-APAS-lime mixture is more than three times that of sand stabilized with cement. The thermal conductivity of sand stabilized with cement and that of sand treated with EM-APAS-lime mixture are 0.1 W/m·K and 0.9–1 W/m·K, respectively. It is confirmed that the heat collection of sand stabilized with EM-APAS-lime outperforms five times over that of sand stabilized with cement. These findings provide admissible evidence that the EM-APAS-lime mixture, which outperforms cement in compressive strength and thermal conductivity, is most suitable for ground improvement binder for GCHP systems.

## 1. Introduction

New renewable energy methods attract considerable attention continuously [1,2,3]. Existing research are associated with natural-source-based energy generation systems that use natural energy sources for obtaining renewable energy [4,5]. Among them, ground-coupled heat pump (GCHP) systems, which are installed underground in which the temperature is maintained constant and may be installed at depths of several tens of meters or more depending on the local ground properties, harvest thermal energy from the underground [6,7,8,9]. GCHP makes use of the heat flow of the region in which it is installed; hence, the efficiency of it depends on the thermal conductivity (TC) of soil surrounding it (i.e., heat pipe). The higher the TC of soil surrounding GCHP systems, the greater the energy efficiency [10]. For certain, the properties of soil surrounding GCHP should be enhanced to protect the heat exchanger(s) and pipe(s) by increasing soil density, hence, resisting against earth pressure. A ground improvement would be recommended for this purpose.

Ground improvement techniques have been developed to solve ground engineering problems. They include several methods such as reinforcement, admixtures or grouting, compaction, and dewatering, etc. [11]. Each has own engineering features and advantages; the designer selects suitable method to accommodate the purpose of improvement. It is well accepted that mixing or grouting with cement is the simplest and advantageous because a significant increase in the soil strength may be achieved within a short period of time (i.e., a month), even if it impacts adversely a little on the environment [12,13,14]. GCHP system makes use of the grouting method injecting cement that is mixed with bentonite to the underground [15]. However, the application of cement does not perfectly lend itself to GCHP systems since the soil mixture surrounding a GCHP may not have appropriate strength and TC. That is why it would be desirable to develop a soil binder exerting higher strength and TC rather than cement.

The grout mixed with cement and bentonite surrounds heat pipe(s) and heat exchanger(s) and blocks the other particles by lowering porosity, hence, guarding the GCHP [16]. The thermal property of a grouting material is involved in energy efficiency, hence, affecting to the number and length of boreholes; the strength property of it affect to stability of the GCHP in situ [17]. Existing studies confirm the epoxy emulsion (EM) and acrylic polymer aqueous solution (APAS) mixture outperforms in strength development as a red clay stabilizer [18,19,20,21]. However, few study considers its energy efficiency and strength when it is mixed with sand for grouting of GCHP system. For certain, it is desirable for a GCHP system to use a material with high TC and strength. Therefore, this study confirms the alternative method, which uses epoxy emulsion (EM) and acrylic polymer aqueous solution (APAS), achieves higher TC over cement. This study investigates the effect of the EM-APAS mixture treatments on the properties of a standard sand used in Korea (i.e., Jumunjin sand). This paper presents the optimal binder composition of EM and APAS for GCHP systems. The optimal solution was determined by evaluating their compressive strength, TC, and durability of the binder. Because sands are always found with fine particles having varying sizes, the standard sand (i.e., Jumunjin sand) is used to control the effect attributed to the variation of sand particle sizes. Simulation output analysis was performed using the heat transfer flow model of GCHP system incorporating experimental results. The optimal solutions obtained by the numerical analysis on the EM-APAS mixture are compared with those on cement.

## 2. Materials and Methods

### 2.1. Materials

#### 2.1.1. Epoxy Emulsion and Acrylic Polymer Aqueous Solution

**Figure 1:**
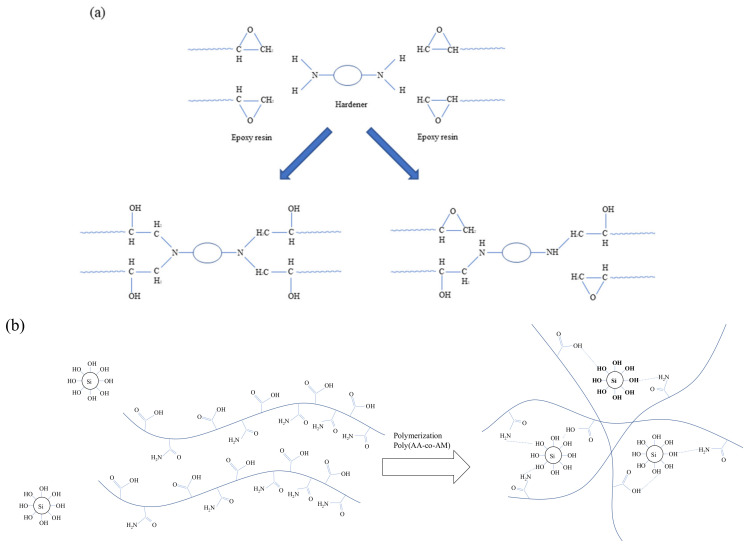
The reactions of (**a**) the epoxy with hardener, and (**b**) APAS chain with the sand.

KEM-101-50 waterborne epoxy manufactured by Kukdo Chemical in Seoul, Korea is used for this study. It contains EM including a hardener (i.e., KH-700, Kukdo Chemical, Seoul, Korea) for the epoxy resin to speed up hardening. The properties of the epoxy and hardener are presented in Table 1 and Table 2. The chemical composition and structure of the EM are not disclosed by the manufacturer due to proprietary reason [21]. However, the hardening reaction of the epoxy with hardener is illustrated on Figure 1a [21]. All EMs used in this study formulated to have the ratio of hardener and epoxy of 2.5:1. The APAS is generated by the reaction mechanism by formulating the ingredients of acrylic acid greater than 99%, 4,4′-azobis (4-cyanovaleric acid) greater than 98%) and ethyl alcohol equal to 94% [22]. Acrylamide and acrylic acid underwent polymerization process through the thermal radical formation of an initiator and formed covalent bonds [20]. The synthesis process of APAS and relationship with APAS and EM are elaborated in detail by Yoon et al. [21]. The curing reaction of the 5 wt% APAS used in the study are shown in Figure 1b [21]. The hydrophilic group of the copolymer was obtained by hydrogen bonding with the surface of the soil particle, and the epoxy penetrated be-tween the copolymers to form IPN (Interpenetrating Polymer Network), hence, strongly agglomerating the soil particle. SEM and apparent density were measured to confirm the role of the copolymer and epoxy [21].

#### 2.1.2. Sand and Cement

**Figure 2:**
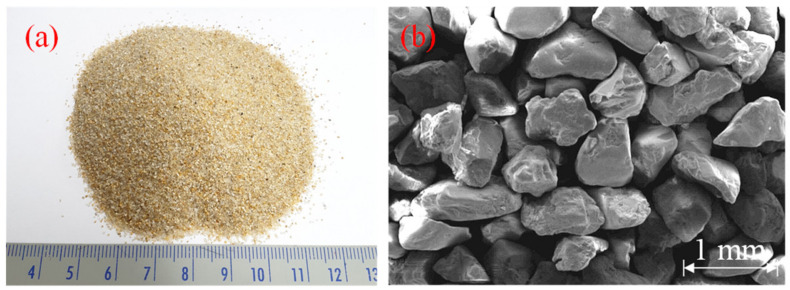
(**a**) Optical photograph and (**b**) microscale SEM image of Jumunjin sand.

Jumunjin sand is classified into poorly graded sand (SP) according to the Unified Soil Classification System as illustrated in the optical photograph and the scanning electron microscopy (SEM, SU8230, Hitachi, Tokyo, Japan) image of it in Figure 2a,b, respectively. Its chemical composition is presented in Table 3. The X-ray fluorescence (S8 Tiger, Bruker, Billerica, MA, USA), which was used for component analysis, confirms that the main constituent of Jumunjin sand is silica (SiO_2_; >87%), and 64% of the particles are in the size range 0.425–0.85 mm. Owing to the high silica content, the SEM image of particles are mainly angular. To compare the effects of the EM and APAS, the cement specimen was manufactured by following Korean Portland cement Standard (KS L 5201) [23]. The cement is Portland cement provided by Ssangyong C&E (Seoul, Korea).

### 2.2. Methods

#### 2.2.1. Sample Preparation

Five types of binder specimen (i.e., EM-treated sand (E), APAS-treated sand (P), EM-APAS mixture sand (EP), lime-containing EP (EPL), and cement specimen (C)) are produced using a cylindrical mold of which diameter and height are of 5 cm and 10 cm, respectively. The weight percentages of sand, EM, APAS, cement and lime are used to control their relative ratio in a specimen preparation. The unit weight of a specimen mold (hereafter mold) is designed to be 15 kN/m^3^. Each mold is manufactured by accommodating five layers each of which is compacted with a rammer applying 2.5 kgf. The compacted specimens are cured in a chamber in which the temperature and humidity are maintained in constant 25 °C and 70%, respectively. The specimens underwent the curing process are demolded after 24 h age. The curing ages and ingredients of the specimens are presented in Table 4. The unconfined compressive strength (UCS) and TC of E type specimens and those of and P type specimens were measured to identify the optimum composition of EP and EPL. The binder ratio of EP type and that of EPL type were determined by consulting the result of E and that of P. Cement ratio was used same amount with EM and P for comparing.

#### 2.2.2. Strength, Thermal Conductivity, Durability Test

The strength was measured by performing the unconfined compression test according to ASTM D 2166 [24] and KS F 2314 [25]. The specimen was pressed with a loading rate of 1 mm/min by moving upward a bottom plate while recording the compression distance and stress. The value of UCS dictates the average of three specimen with an error less than 5%. The TC was measured by taking the procedure defined in ASTM D 5334 [26] using a needle-probe device in which two sensors (i.e., heat sensor and measuring sensor) implemented for soil and soft rock. The former obtains the amount of heat generated by the heat source attaching the heat sensor; the latter the transient temperature increases around the measuring sensor. The slake durability was measured by taking Franklin and Chandara [27], and ASTM D 4644 [28]. After soaking a sieve drum in water, ten pieces of a dried rock, which has an equidimensional shape and an approximately 50 g weight, were put in the drum. The soaked pieces were worn away while rotating the sieve drum at a speed of 20 rev/min. The durability index is calculated by dividing the difference which is obtained by subtracting the dry weight of the rock pieces remained after rotating the drum from the dry weight of the rock pieces before being soaked by the initial weight of the pieces. After conducting the test twice for each specimen, the durability index obtained by the second test was accepted as the representative value of the specimen by following ASTM D 4644 [28].

#### 2.2.3. Heat Transfer Fluid Dynamic Model of GCHP Systems

**Figure 3:**
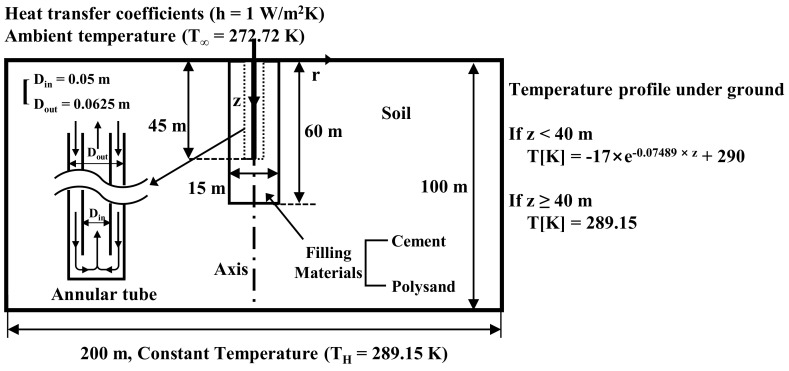
A schematic simulation model of the thermal performance of GCHP.

Controlled experiments were performed by fixing the inter diameter, outer diameter, and length of the annular tube of GCHP, the velocity of water incoming into the inlet of annular tube, the diameter of borehole, and the length of borehole to 0.05 m, 0.0625 m, 45 m, 0.1 m/s, 15 m, and 60 m, respectively, in the simulation model shown in Figure 3. The manipulated variable is the type of material (i.e., sand-cement grout or sand-EM-APAS mixture) to be filled into the borehole. By controlling most of the variables excepting the type of material, the system analysis is simplified into a two-dimensional axisymmetric problem which compare the thermal performance of sand stabilized with cement and that of sand with EM-APAS. Given the solution space (i.e., heat-collecting domain) of which the radius is 100 m and the depth from the ground surface is 100 m, the temperature profile varies along the depth.

The ambient temperature and the heat transfer coefficient between the air and ground are set to 272.72 K and 1 W/m^2^ K, respectively, by considering the continuity of temperature and heat flux at the ground surface. The temperature profile underground is obtained by approximating the result of Al-Sarkhi et al. [29] as shown in Equations (1) and (2).

T[K] = −17exp(−0.07489z) + 290 (z < 40 m)(1)

T[K] = 289.15 (z ≥ 40 m)(2)

## 3. Results and Discussion

### 3.1. Performance of Epoxy Emulsion and Polymer Solution

**Figure 4:**
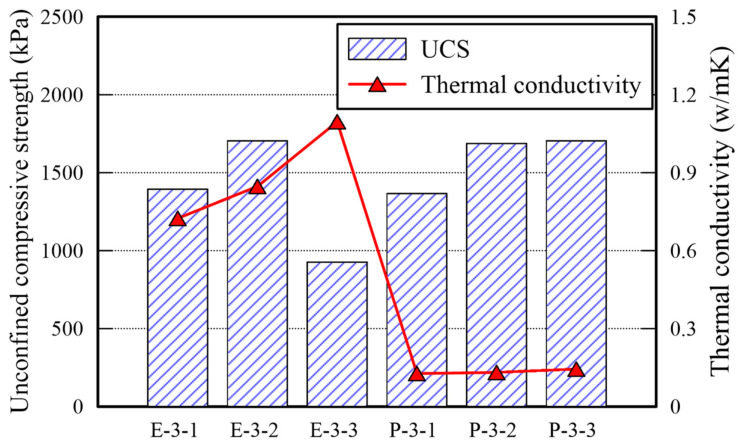
UCS and TC for E and P type specimens.

The UCS and TC of E and P type specimens are shown in Table 5 and Figure 4. The UCS values of both type specimens exceed 1000 kPa given a binder ratio of 4%; the strengths of them are similar given binder ratios of 4% and 8%. In addition, given a binder ratio of 12%, the UCS value of the specimen treated with EM is halved; that of the specimen treated with APAS increases slightly. The water content of both specimens exceeds 1% after 3 days curing. It confirms that 3 days curing is not sufficient for hardening given the 12% binder ratio. It may not be possible to keep the water content intact at underground owing to several factors (i.e., precipitation, earth pressure, underground water, and seismic activity). That is why the early strength development of soil under the ground by treating chemically is important as done in every soil improvement binder. It is found that the optimal binder ratio of EM and APAS is 8%. Given 8% ratio, the early strength arrives at a sufficient value in a short period.

**Figure 5:**
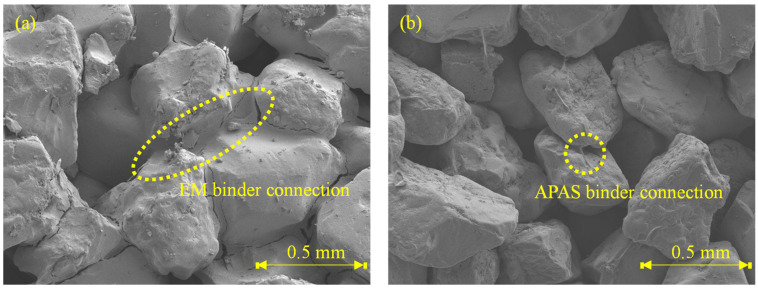
Microscale images obtained by SEM analysis of (**a**) EM- and (**b**) APAS-treated sand.

The TC of EM-treated sand is greater than that of APAS-treated sand by over six times. The difference between these two is apparent in the SEM analysis images each of which presents the microstructure of each specimen given a binder ratio of 8% as shown in Figure 5. Indeed, the extent of connection among sand particles in each specimen is remarkably different. The bond of sand particles in E type specimen cover a wider region compared with that in P type specimen. The more the number of connection bonds among sand particles, the more effectively transfers heat. The reason is that air has considerably lower TC than TC of other materials [30]. TC of air usually has around 0.0262–0.0549 W/m·K [31]. Enough number of connection bonds. Given a specimen treated with 8% APAS binder ratio, the polymer molecular and water contents are 0.4% and 7.6%, respectively, because 5 wt% of APAS was used in this study. SEM analysis confirms that the polymer manifests high strength with a low molecular weight. Indeed, the outputs associated with TC are significantly different from those associated with UCS.

### 3.2. Epoxy Emulsion-Polymer Solution Mixture

**Figure 6:**
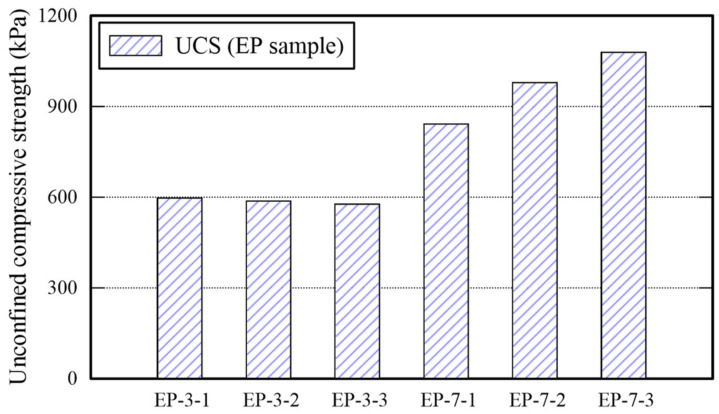
Unconfined compressive strength of EP specimens.

All EP-3-x specimens cured for 3 days are collapsed as shown in the outputs of EP specimen presented in Table 6 given the compression. It confirms that 3 days curing is not sufficient to harden the EM- and APAS-treated sand specimen, because there were sand particles enclosed with water inside of the specimens during the compression test and the water contents of the sample was over 2 times than those of EP-7-x. For certain, the surface of each specimen was completely dried before applying compressive strength. However, there were sand particles enclosed with water from which the failure starts during compression inside of the specimens. All EP specimens (i.e., EP-7-x series) cured for 7 days were completely dried, hence, retaining less than 0.5% water content. The UCS associated with these EP specimens increase as the APAS ratio increase. Note that the maximum UCS value (i.e., 1079 kPa) of EP specimens is lower than those of E and P specimens, hence, decreasing the strength when the EM-APAS mixture is used. The rational for this phenomenon may be that the moisture retained in the APAS disperses the EM and reduce the area over which particles have connected each other. It appears that high polymer solution rate makes the epoxy treated soil sample weak [20]. The variability of the UCS is shown in Figure 6.

**Figure 7:**
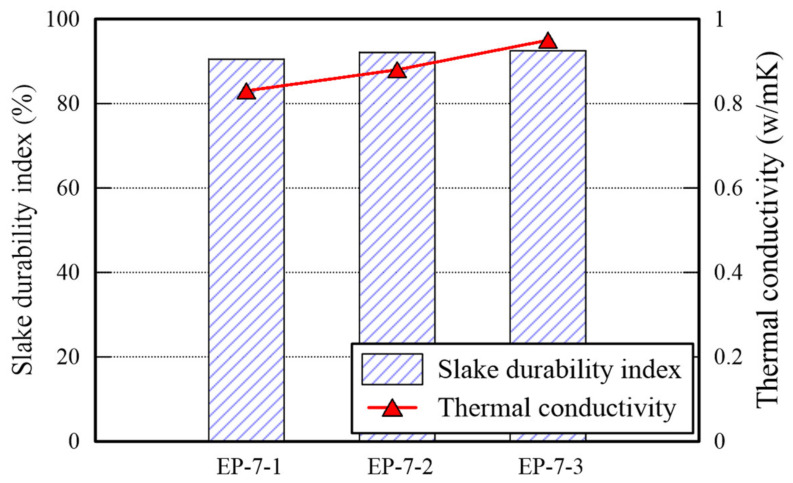
TC and SDI of EP specimens.

The TC and slake durability index (SDI) of EP specimens are presented in Figure 7. The TC of it is in the range of 0.8–1.0 W/m·K which is significantly higher than that of P specimens, but is similar with that of E specimens. It appears that many voids in the specimen shown in Figure 5a are filled by mixing EM. However, the TC of EP type underperform than that of E type. Thus, The UCS and TC tests provide admissible evidence that the EM-APAS mixture is less efficient than EM. The SDI is over 90%. The higher the durability index generally, the higher the strength of a specimen. The strength of EP specimens cured for 7 days is over 800 kPa, hence, being the durability index over 90%. Given a binder having low resistance to water, the durability index decreases significantly when the binder which is a part of the specimens is dissolved after immersing them into water. It confirms that the EP specimens has high resistance to abrasion attributed to friction between particles and to water penetration.

### 3.3. Epoxy Emulsion-Polymer Solution-Lime Mixture

EP specimens achieves high durability index and acceptable UCS and TC. However, their strength is not justified for the usage in deep underground. The experiments obtained by E, P, EP specimens confirm that the strength of EM- treated sand and that of APAS-treated sand are affected by the amount of moisture mixed in the binders. Therefore, lime, which has been used traditionally for improving the strength of the ground, is added to dewater the moistures by activating hydration reaction. The experiment outputs obtained by EM-, APAS-, and lime-treated sand are shown in Table 7.

**Figure 8:**
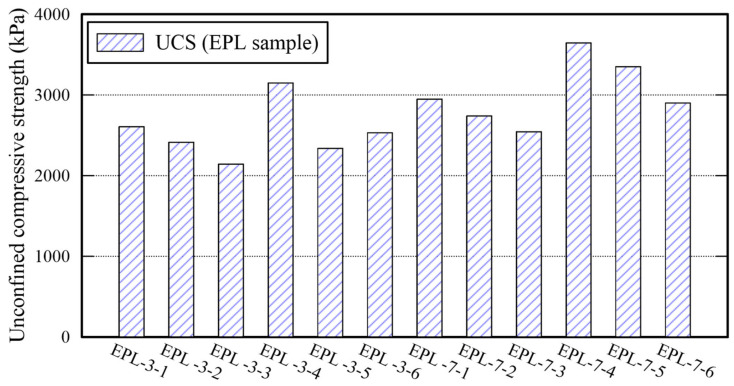
Unconfined compressive strength of EPL specimens.

**Figure 9:**
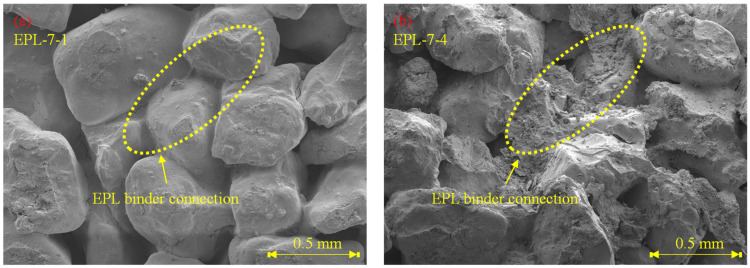
SEM analysis images of EPL specimen: (**a**) EPL-7-1 and (**b**) EPL-7-4.

The UCS of EPL specimens are more or less high than those of E, P, and EP specimens as shown in Figure 8. That of EPL gets over 2000 kPa after 3 days curing. For example, that of EPL-3-4 is 3148 kPa. It increases as the curing time is elapsed from 3 to 7 days. The significant increase in UCS is attributed to the chemical reactions obtained by lime dewatering the moisture from the binder and filling the voids among the sand particles. Because Ca(OH)_2_ are made when lime contact with water [32]. The void filling is confirmed by an SEM analysis image as shown in Figure 9. Figure 9 show the corresponding SEM image of EPL-7-1 with 3% lime content and that of EPL7-4 with 6% lime content, respectively, while holding 1% APAS. Their binder connections supply wider and thicker structure than those obtained by E and P specimens shown in Figure 5. Indeed, it is found that the microcracks at the binder bond are disappeared. In addition, it appears that the lime particles among sand particles increase significantly when the lime content increases from 3% to 6% at Figure 9.

**Figure 10:**
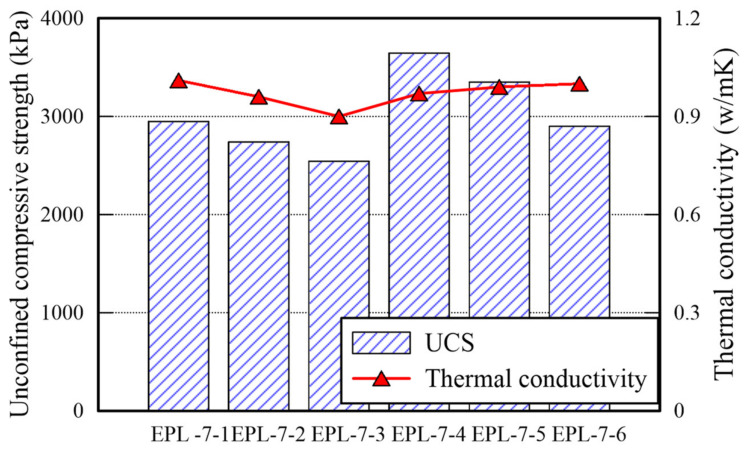
TC and SDI of EPL specimen.

The TC and SDI of each EPL specimen are presented in Figure 10. The TC is over 0.9, particularly, the specimen retaining 6% lime gets over 0.97. The difference between TC of EP specimens and that of E specimens is minimal. However, the TC of EPL specimens increase since the added lime fills the voids inside of the specimens. Because TC of air is lower than that of other soil and that of clay materials [30], The replacement of the airs occupying the voids with lime contributes transferring heat more efficiently rather than the airs does. However, the more lime is added, the lower the SDI decreases. Given impact to the cylinder used for the slake durability test, the lime particles fell off more easily rather than sand particles do. The reason is that the size of lime particles is smaller than that of sand particles. The EPL-7-4 and EPL-7-5 specimens outperform in the three parameters (i.e., UCS, TC, and SDI). Therefore, adding 6% of lime enhances the strength of the specimen significantly. It is confirmed that 6:1.0 or 6:1.5 of lime-APAS rate make lime optimally react well, but 6:2.0 of lime-APAS rate reduces UCS and SDI.

### 3.4. Comparison with Sand Stabilized with Cement

**Figure 11:**
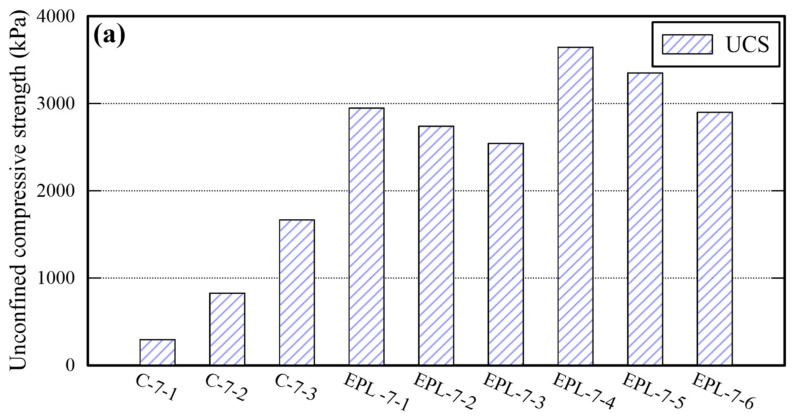

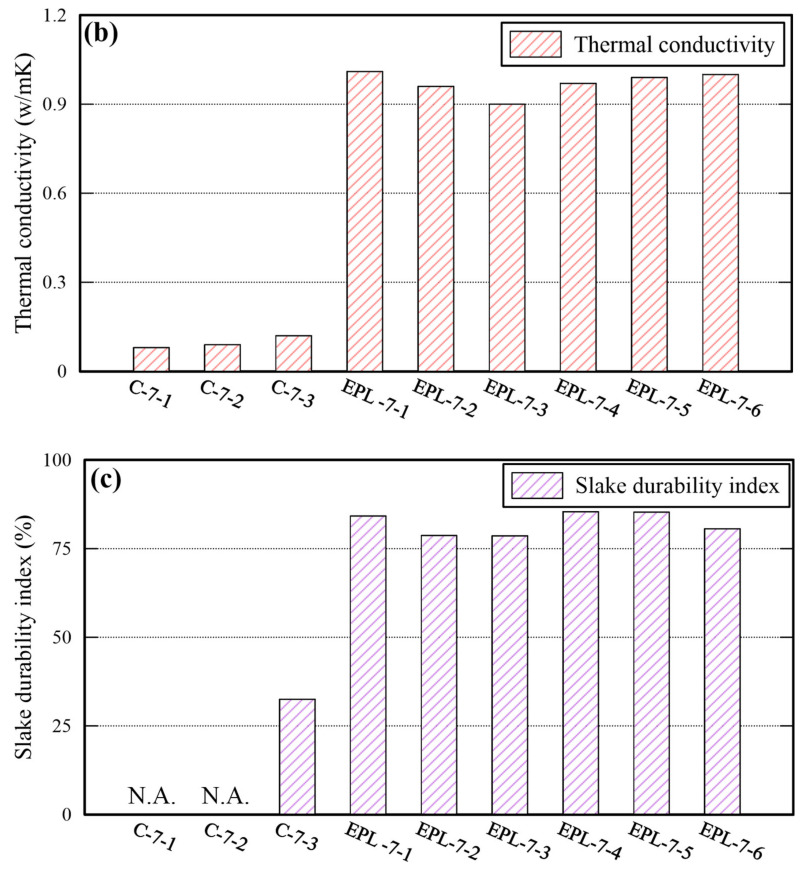
Comparison of the UCS (**a**), TC (**b**), and SDI (**c**) between cemented and EPL specimens.

Sand cylinders stabilized with cement were prepared to compare their properties with those of EPL specimens. The percentage of cement content is controlled by taking the identical method used for the other binders (i.e., EM, APAS, and lime) and 15% of water was added. The properties of sand cylinders are shown in Table 8. Given 12% cement content, the maximum UCS of them is 1666 kPa which is similar to that of E and that of P specimens, and lower than that of EPL specimens. For certain, the TC and SDI of sand cylinders stabilized with cement are lower than those of each binder used in this study. Particularly, the SDI of the sand cylinders, which were stabilized with 4% and 8% cement, could not even measured because all pieces of specimens were worn away and disappeared during the test. The properties deviation between cement stabilized sand and EPL specimens are shown in Figure 11. The cemented stabilized sand specimens underperform significantly in the unconfined compressive strength, TC, and SDI than EPL specimens. The UCS and SDI of cemented stabilized sand specimens may be improved to be comparable to those of the EPL sample by increasing the cement content. The reason is that those values increase only slightly even though the cement content increases. However, the TC of them may not be easily reach to the corresponding values of EPL specimens. These phenomena provide the admissible evidence that EM and APAS are more suitable for ground binders of GCHP systems than cement. For certain, high TC value of ground is required to the GCHP system installed in the ground. At the same amount of cement and EPL, TC of EPL showed over 8 times higher than that of cement.

### 3.5. Simulation Output Analysis of the Heat Transfer Fluid Model

Thermal performance of each sand stabilized with different material (i.e., EPL-7-5 and C-7-3) were estimated by computing the thermal conductivity of them using the material properties shown in Table 9.

**Figure 12:**
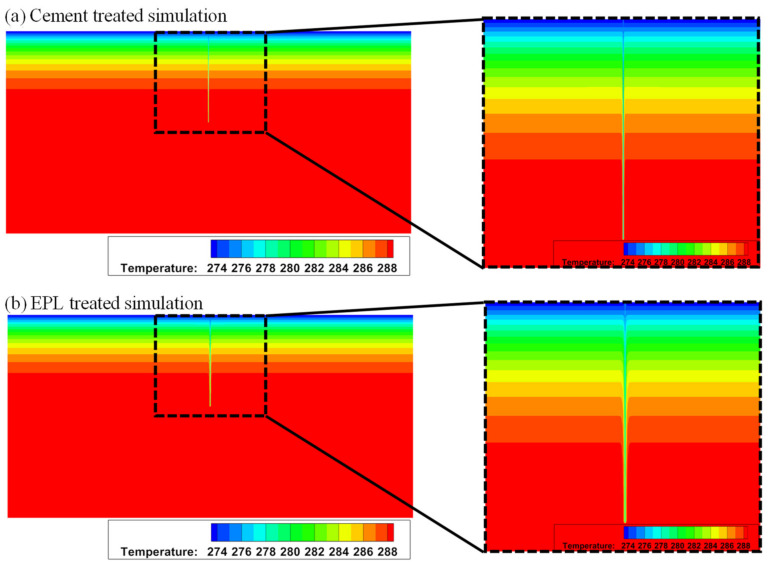
Isotherms under the ground stabilized with (**a**) cement and (**b**) EPL.

When the sand stabilized with cement and that stabilized with EM-APAS-lime are used for back-filling material, the isotherms of them are compared in the solution space (i.e., heat-collecting domain). The thermal diffusion of the borehole, which is back-filled with sand stabilized with EPL, is much bigger than the others as shown in the zoom-in view presented in the right-hand column of Figure 12.

**Figure 13:**
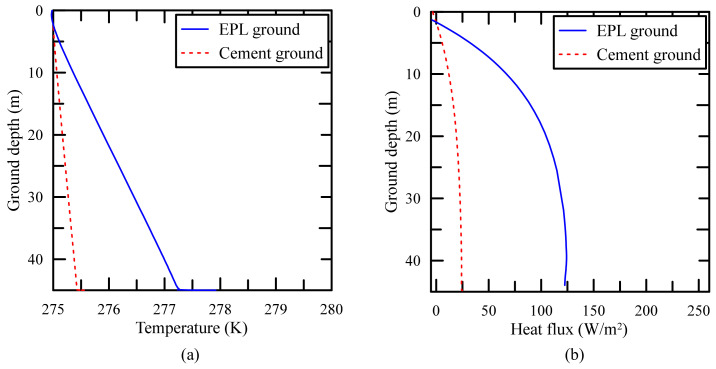
Comparison of (**a**) temperature profiles and (**b**) heat flux profiles for different filling materials.

The distribution of temperature and that of heat flux on the cylindrical side wall of GCHP are shown in Figure 13. The temperature and the heat flux around the side wall of GCHP are high and large, respectively, due to the high thermal conductivity of EPL binder as shown in Figure 13b.

Given the ground stabilized with cement and that stabilized with EPL, respectively, the water temperature increase and heat collection via the cylindrical GCHP are compared as shown in Table 10. It is confirmed that the ground stabilized with EPL outperforms by five times in heat collection performance over the ground stabilized with cement.

## 4. Conclusions

The study identifies the best fit ratio which is most favorably associated with ground binders using epoxy emulsion (EM) and acrylic polymer aqueous solution (APAS) for ground-coupled heat pump (GCHP) systems. After evaluating the performance (i.e., unconfined compression, thermal conductivity, and slake durability) of Jumunjin sand specimens stabilized with EM, APAS, EM-APAS mixture, or EM-APAS-lime mixture, the research findings are obtained as follows:

First, the best UCS and TC of EM-treated sand specimens are 1734 kPa and 1.10 W/m·K, respectively. Those of APAS-treated sand specimens are 1704 kPa and 0.14 W/m·K, respectively. EM- and APAS-treated sand get similar compressive strength. It confirms that APAS-treated sand is favorable to EM-treated sand in the TC, because the TC of the former is about eight times that of the latter. Second, the UCS and TC of EP specimens, which is made of EM-APAS mixture, underperform compared to those of EM-treated sand specimens. However, the SDI of EP is about 90% higher than that of EM-treated sand specimens. In addition, the UCS of EP may increase to over 3000 kPa by adding lime. Third, the lime-added EP (EPL) specimens outperform in the UCS, TC, and slake durability tests. The best UCS, TC, and SDI are 3645 kPa, 1.00 W/m·K and 85.4%, respectively. These outstanding performances are attributed to the hydration reaction by which the lime removes the moisture in the EM and APAS and forms connecting bonds. Fourth, the UCS, TC, and SDI of EPL specimens are higher than those of sand stabilized with cement. EPL specimens outperform in strength, thermal efficiency, and durability, when the EPL binder and cement are separately used as sand binders. In addition, the heat flow numerical simulation confirms ground stabilized with EPL outperforms in heat collection five times over ground stabilized with cement. It is found that EM and APAS is more effective ground binder for GCHP system than cement.

## Figures and Tables

**Table 1:** Physical properties of the epoxy.

Model	EEW ^1^ (g/eq)	Viscosity (cps@25 °C)	Nonvolatile Content (wt%)
KEM-101-50	450–550	1000–10,000	47

^1^ EEW denotes “epoxy equivalent weight”.

**Table 2:** Physical properties of the hardener.

Model	TAV ^1^ (mg KOH/g)	Viscosity (cps@25 °C)	AHEW ^1^ (g/eq)	Nonvolatile Content (wt%)
KH-700	190–250	3000–10,000	170	80

^1^ TAV and AHEW denote “total amine value” and “amine hydrogen equivalent weight”, respectively.

**Table 3:** Chemical composition of Jumunjin sand.

	SiO_2_	Al_2_O_3_	K_2_O	NA_2_O	Fe_2_O_3_	CaO	BaO	Cl
**Component (%)**	87.70	6.61	4.03	0.76	0.25	0.11	0.09	0.07

**Table 4:** Ingredients and test parameters of the specimen.

Type	Binder	Binder Ratio (%)	Curing Time (Days)	Test Parameter
E	EM	4, 8, 12	3	Unconfined compressive strength Thermal conductivity SEM analysis
P	APAS	4, 8, 12		
EP	EM+APAS	EM: 8 APAS: 1, 1.5, 2	3	Unconfined compressive strength Thermal conductivity ^1^ Slake durability index ^1^
			7	
EPL	EM+APAS+Lime	EM: 8 APAPS: 1, 1.5, 2 Lime: 3, 6	3	Unconfined compressive strength Thermal conductivity ^1^ Slake durability index ^1^ SEM analysis
			7	
C	Cement	4, 8, 12	3	Unconfined compressive strength Thermal conductivity ^1^ Slake durability index ^1^
			7	

^1^ Thermal conductivity and slake durability tests for EP and EPL were conducted only on samples cured for seven days.

**Table 5:** Experiment outputs associated with E and P type specimens.

Type	Sample Code	Curing Time (Days)	Binder Ratio (%)	Dry Unit Weight (kN/m^3^)	Water Content (%)	Unconfined Compressive Strength (kPa)	Thermal Conductivity (W/m·K)
E	E-3-1	3	4	15.06	0.93	1394	0.724
	E-3-2		8	15.50	0.80	1734	0.847
	E-3-3		12	15.79	1.63	926	1.096
P	P-3-1	3	4	14.32	0.19	1366	0.127
	P-3-2		8	14.42	0.17	1687	0.131
	P-3-3		12	14.42	1.31	1704	0.144

**Table 6:** Experiment outputs for EP type specimens.

Type	Sample Code	Curing Time (Days)	EM (%)	APAS (%)	Dry Unit Weight (kN/m^3^)	Water Content (%)	Unconfined Compressive Strength (kPa)	Thermal Conductivity (W/m·K)	Slake Durability Index (%)
EP	EP-3-1	3	8	1.0	15.44	0.94	597	-	-
	EP-3-2			1.5	15.55	0.96	587		
	EP-3-3			2.0	15.66	1.16	577		
	EP-7-1	7		1.0	15.45	0.39	842	0.83	90.5
	EP-7-2			1.5	15.57	0.35	979	0.88	92.1
	EP-7-3			2.0	15.45	0.42	1079	0.95	92.5

**Table 7:** Experiment outputs for EP type specimens.

Type	Sample Code	Curing Time (Days)	EM (%)	APAS (%)	Lime (%)	Dry Unit Weight (kN/m^3^)	Water Content (%)	Unconfined Compressive Strength (kPa)	Thermal Conductivity (W/m·K)	Slake Durability Index
EPL	EPL-3-1	3	8	1.0	3	15.56	0.28	2606	-	-
	EPL-3-2			1.5		15.72	0.48	2413		
	EPL-3-3			2.0		15.67	0.46	2142		
	EPL-3-4			1.0	6	15.63	0.35	3148		
	EPL-3-5			1.5		15.55	0.90	2338		
	EPL-3-6			2.0		15.57	0.68	2531		
	EPL-7-1	7		1.0	3	15.52	0.42	2948	1.01	84.2
	EPL-7-2			1.5		15.51	0.41	2740	0.96	78.7
	EPL-7-3			2.0		15.44	0.48	2543	0.90	78.6
	EPL-7-4			1.0	6	15.63	0.36	3645	0.97	85.4
	EPL-7-5			1.5		15.58	0.42	3350	0.99	85.3
	EPL-7-6			2.0		15.79	0.49	2900	1.00	80.6

**Table 8:** Experiment outputs for the cemented specimens.

Type	Sample Code	Curing Time (Days)	Cement Content (%)	Dry Unit Weight (kN/m^3^)	Water Content (%)	Unconfined Compressive Strength (kPa)	Thermal Conductivity (W/m·K)	Slake Durability Index
C	C-3-1	3	4	14.94	1.01	215	-	-
	C-3-2		8	15.74	1.44	587		
	C-3-3		12	15.95	2.19	975		
	C-7-1	7	4	14.44	0.45	294	0.08	N.A. ^1^
	C-7-2		8	15.06	0.67	826	0.09	N.A. ^1^
	C-7-3		12	15.45	1.01	1666	0.12	32.5

^1^ N.A. means ‘None available’.

**Table 9:** Thermal fluidic properties of ground material.

Material	Thermal Conductivity (W/m·K)	Specific Heat (J/kg·K)	Unit Weight (kN/m^3^)	Viscosity (kg/m·s)
Cement-sand	0.12	1040	14.4	-
EPL-sand	0.99	880	18.0	-
Ground soil	0.22	760	13.0	-
Water	0.6	4182	10.0	0.01

**Table 10:** Temperature increase and heat collection for the grounds stabilized with EPL and cement.

	Temperature Change (K)	Heat Collection Rate (W)
Ground stabilized with EPL	2.057	915.746
Ground stabilized with cement	0.405	183.8

## Data Availability

Data sharing not applicable to this article as no datasets were generated or analysed during the current study.

## Abbreviations

The following symbols are used in this article:

APAS	acrylic polymer aqueous solution
E	EM-treated sand
EM	epoxy emulsion
EP	EM-APAS mixture sand
EPL	lime-containing EP (EPL)
GCHP	ground-coupled heat pump
P	APAS-treated sand
SDI	slake durability index

## References

[1] Bull S.R. (2001). Renewable Energy Today and Tomorrow. Proc. IEEE.

[2] Ellabban O., Abu-Rub H., Blaabjerg F. (2014). Renewable Energy Resources: Current Status, Future Prospects and their Enabling Technology. Renew. Sustain. Energy Rev..

[3] Demirbaş A. (2006). Global Renewable Energy Resources. Energy Sources Part A Recover. Util. Environ. Eff..

[4] Danish, Ulucak R., Khan S.U.-D. (2019). Determinants of the Ecological Footprint: Role of Renewable Energy, Natural Resources, and Urbanization. Sustain. Cities Soc..

[5] Danish, Baloch M.A., Mahmood N., Zhang J.W. (2019). Effect of Natural Resources, Renewable Energy and Economic Development on CO_2_ Emissions in BRICS Countries. Sci. Total Environ..

[6] Zhai X., Qu M., Yu X., Yang Y., Wang R. (2011). A Review for the Applications and Integrated Approaches of Ground-Coupled Heat Pump Systems. Renew. Sustain. Energy Rev..

[7] Yi M., Hongxing Y., Zhaohong F. (2008). Study on Hybrid Ground-Coupled Heat Pump Systems. Energy Build..

[8] Bernier M.A. (2001). Ground-Coupled Heat Pump System Simulation/Discussion. ASHRAE Trans..

[9] Noorollahi Y., Saeidi R., Mohammadi M., Amiri A., Hosseinzadeh M. (2018). The Effects of Ground Heat Exchanger Parameters Changes on Geothermal Heat Pump Performance—A Review. Appl. Therm. Eng..

[10] Lee C., Park M., Min S., Kang S.-H., Sohn B., Choi H. (2011). Comparison of Effective Thermal Conductivity in Closed-Loop Vertical Ground Heat Exchangers. Appl. Therm. Eng..

[11] Kirsch K., Bell A. (2012). Ground Improvement.

[12] Kitazume M., Terashi M. (2013). The Deep Mixing Method.

[13] Zebovitz S., Krizek R.J., Atmatzidis D.K. (1989). Injection of Fine Sands with Very Fine Cement Grout. J. Geotech. Eng..

[14] Güllü H., Canakci H., Al Zangana I.F. (2017). Use of Cement Based Grout with Glass Powder for Deep Mixing. Constr. Build. Mater..

[15] Allan M., Philippacopoulos A. Performance Characteristics and Modelling of Cementitious Grouts for Geothermal Heat Pumps. Proceedings of the World Geothermal Congress.

[16] Mikkelsen P.E. (2002). Cement-Bentonite Grout Backfill for Borehole Instruments. Geotech. News.

[17] Witte H.J., Van Gelder G.J., Spitler J.D. (2002). In Situ Measurement of Ground Thermal Conductivity: A Dutch Perspective. ASHRAE Trans..

[18] Kim J., Choi H., Rye H.M., Yoon K.B., Lee D.E. (2021). A Study on the Red Clay Binder Stabilized with a Polymer Aqueous Solution. Polymers.

[19] Kim J., Choi H., Yoon K.-B., Lee D.-E. (2020). Performance Evaluation of Red Clay Binder with Epoxy Emulsion for Autonomous Rammed Earth Construction. Polymers.

[20] Park S.-S., Lee J.-S., Yoon K.-B., Woo S.-W., Lee D.-E. (2021). Application of an Acrylic Polymer and Epoxy Emulsion to Red Clay and Sand. Polymers.

[21] Yoon K.-B., Ryu H.M., Lee G.H., Gopalan A.I., Sai-Anand G., Lee D.-E. (2021). Enhanced Compressive Strength of Rammed Earth Walls Stabilized with Eco-Friendly Multi-Functional Polymeric System. Renew. Sustain. Energy Rev..

[22] Rye H.M., Son N.R., Park J.Y., Yi C.Y., Lee D.E., Yoon K.B. (2019). Acrylic Copolymer as Soil Stabilzer for Improving Compresive Strength of Red Clay Soil. Polymers.

[23] (2006). Portland Cement.

[24] (2000). Standard Test Method for Unconfined Compressive Strength of Cohesive Soil.

[25] (2018). Standard Test Method for Unconfined Compression Test of Soils.

[26] (2008). Standard Test Method for Determination of Thermal Conductivity of Soil and Soft Rock by Thermal Needle Probe Procedure.

[27] Franklin J.A., Chandra R. (1972). The slake-durability test. Int. J. of Rock Mech. Min. Sci. Geomech. Abstr..

[28] (2008). Standard Test Method for Slake Durability of Shales and Other Similar Weak Rocks.

[29] Al-Sarkhi A., Abu-Nada E., Nijmeh S., Akash B. (2008). Performance Evaluation of Standing Column Well for Potential Application of Ground Source Heat Pump in Jordan. Energy Convers. Manag..

[30] Dafalla M.A., Samman A. (2016). Soil and Backfill Material of Environmental Friendly Thermal Properties. GEOMATE J..

[31] Moskalenko A., Kozhevnikov A. (2016). Estimation of Gas Turbine Blades Cooling Efficiency. Procedia Eng..

[32] Liu H. (2018). Novel Approach on Reduction in GHG Emissions from Sludge Lime Stabilization as an Emergent and Regional Treatment in China. Sci. Rep..

